# Human Embryonic Stem Cells Differentiated to Lung Lineage-Specific Cells Ameliorate Pulmonary Fibrosis in a Xenograft Transplant Mouse Model

**DOI:** 10.1371/journal.pone.0033165

**Published:** 2012-03-28

**Authors:** Ena Ray Banerjee, Michael A. Laflamme, Thalia Papayannopoulou, Michael Kahn, Charles E. Murry, William R. Henderson

**Affiliations:** 1 Center for Allergy and Inflammation and Institute for Stem Cell and Regenerative Medicine, Department of Medicine, University of Washington, Seattle, Washington, United States of America; 2 Center for Cardiovascular Biology and Institute for Stem Cell and Regenerative Medicine, Department of Pathology, University of Washington, Seattle, Washington, United States of America; 3 Division of Hematology and Institute for Stem Cell and Regenerative Medicine, Department of Medicine, University of Washington, Seattle, Washington, United States of America; 4 Center for Stem Cell and Regenerative Medicine, Department of Biochemistry and Molecular Biology, University of Southern California, Los Angeles, California, United States of America; University of Pittsburgh, United States of America

## Abstract

**Background:**

Our aim was to differentiate human (h) embryonic stem (ES) cells into lung epithelial lineage-specific cells [i.e., alveolar epithelial type I (AEI) and type II (AEII) cells and Clara cells] as the first step in the development of cell-based strategies to repair lung injury in the bleomycin mouse model of idiopathic pulmonary fibrosis (IPF). A heterogeneous population of non-ciliated lung lineage-specific cells was derived by a novel method of embryoid body (EB) differentiation. This differentiated human cell population was used to modulate the profibrotic phenotype in transplanted animals.

**Methodology and Principal Findings:**

Omission or inclusion of one or more components in the differentiation medium skewed differentiation of H7 hES cells into varying proportions of AEI, AEII, and Clara cells. ICG-001, a small molecule inhibitor of Wnt/β-catenin/Creb-binding protein (CBP) transcription, changed marker expression of the differentiated ES cells from an AEII-like phenotype to a predominantly AEI-like phenotype. The differentiated cells were used in xenograft transplantation studies in bleomycin-treated Rag2γC^−/−^ mice. Human cells were detected in lungs of the transplanted groups receiving differentiated ES cells treated with or without ICG-001. The increased lung collagen content found in bleomycin-treated mice receiving saline was significantly reduced by transplantation with the lung-lineage specific epithelial cells differentiated from ES cells. A significant increase in progenitor number was observed in the airways of bleomycin-treated mice after transplantation of differentiated hES cells.

**Conclusions:**

This study indicates that ES cell-based therapy may be a powerful novel approach to ameliorate lung fibrosis.

## Introduction

The pulmonary system is composed of a variety of epithelial cell populations residing in distinct anatomical locations. Of these, the alveolar epithelial gas exchange surface consists of two cell types, the type I and type II pneumocytes, also known as alveolar epithelial type I and type II (AEI and AEII) cells, that comprise ∼95% and 5% respectively of the alveolar lining area [1]. AEI cells, important in the regulation of alveolar fluid balance [2], are branched cells with cytoplasm extremely attenuated for gas exchange [3]. AEII cells are cuboidal cells situated between AEI cells, and contain characteristic lamellar bodies and apical microvilli [3]. Functions of AEII cells include the secretion and reuptake of pulmonary surfactant [4], regulation of alveolar fluid, and synthesis of immunomodulatory proteins [e.g., surfactant protein (SP)-A, SP-D] important for host defense [5]. The non-ciliated columnar Clara cells [6] constitute the majority of the bronchiolar and terminal bronchiolar epithelium. Clara cells actively divide and differentiate to form ciliated cells, secrete glycosaminoglycans that are major component of the extracellular matrix (ECM), and metabolize airborne toxins by cytochrome P-450 enzymes present in their smooth endoplasmic reticulum [7].

In many life-threatening pulmonary diseases, such as acute lung injury, acute respiratory distress syndrome (ARDS), cystic fibrosis, and idiopathic pulmonary fibrosis (IPF) [8]–[10], endothelial cells and AEI cells are sites of initial damage. As a result, interstitial edema occurs, and increased deposition of ECM proteins such as collagen, laminin, and fibronectin in the lungs resulting in pulmonary fibrosis and loss of the gas exchange surface. For lung injury repair, AEII cells or other lung progenitor cells may replace lost AEI cells to re-establish the thin barrier necessary for efficient gas exchange in the alveolar milieu [11].

Human embryonic stem (hES) cells are a potential source of cells for cell-based therapy in degenerative diseases where there is progressive loss of functional tissue. Cellular replacement therapy or regeneration of lost tissue may potentially reinstate normal tissue structure and function [12], [13]. For tissue replacement therapy to be feasible, sufficient numbers of lung lineage-specific cells need to be engineered *in vitro* for transplantation. A key regulator of stem cell self-renewal with important effects on both cell proliferation and differentiation is the Wnt/β-catenin signaling pathway. Through this canonical Wnt signaling pathway, β-catenin increases in the nucleus and forms a complex with T cell factor (TCF)/lymphoid enhancer factor-1 (LEF-1) transcription factors that are differentiately modulated by Creb-binding protein (CBP) and p300 co-activators. An increase in β-catenin/CBP-mediated transcription by selectively inhibiting β-catenin/p300-mediated transcription maintains stem cell pluripotency, whereas blockade of β-catenin/CBP signaling facilitates β-catenin/p300-mediated transcription and cell differentiation [14]–[16].

Our aim was to differentiate hES cells into lung epithelial lineage-specific cells (i.e., AEI, AEII, and Clara cells) and develop a cell-based strategy in order to repair lung injury in a mouse model of IPF. Previous work [17]–[20] has demonstrated differentiation steps to AEII cells from murine ES cells and the hES H1 cell line. Bleomycin, an anti-neoplastic drug that causes lung fibrosis as a side effect in patients, has been employed in mouse models to induce pulmonary fibrosis. DNA damage by bleomycin induces apoptosis of epithelial cells with loss of AEI cells in the alveolus accompanied by interstitial deposition of collagen and other ECM proteins. It is thought that AEII cells contribute to the repair of the injured lungs by an initial limited proliferation followed by differentiation to AEI cells and Clara cells. Using the selective small molecule blocker ICG-001 that inhibits the β-catenin/CBP interaction without blocking the β-catenin/p300 interaction, we have recently demonstrated a causal role for aberrant Wnt/β-catenin-mediated transcription in development and progression of pulmonary fibrotic disease in the bleomycin mouse model [21].

In this study, we successfully differentiated hES H7 cells in culture into non-ciliated lung lineage-specific cells with intracellular and surface protein markers and morphology characteristic of AEI cells, AEII cells, and Clara cells. The selective β-catenin/CBP inhibitor ICG-001 facilitated the induction/differentiation of AEII cells to AEI cells. We found the differentiated stem cells to home to small airways of mouse lung with bleomycin-induced lung fibrosis in a xenograft transplantation model. Engraftment of the human cells was accompanied by marked reduction of the increased collagen content of the injured murine lungs demonstrating the potential role of hES cell therapy in amelioration of pulmonary fibrosis.

## Results

### Differentiation of hES Cells is Accompanied by Sequential Downregulation of Pluripotent Markers

**Figure 1:**
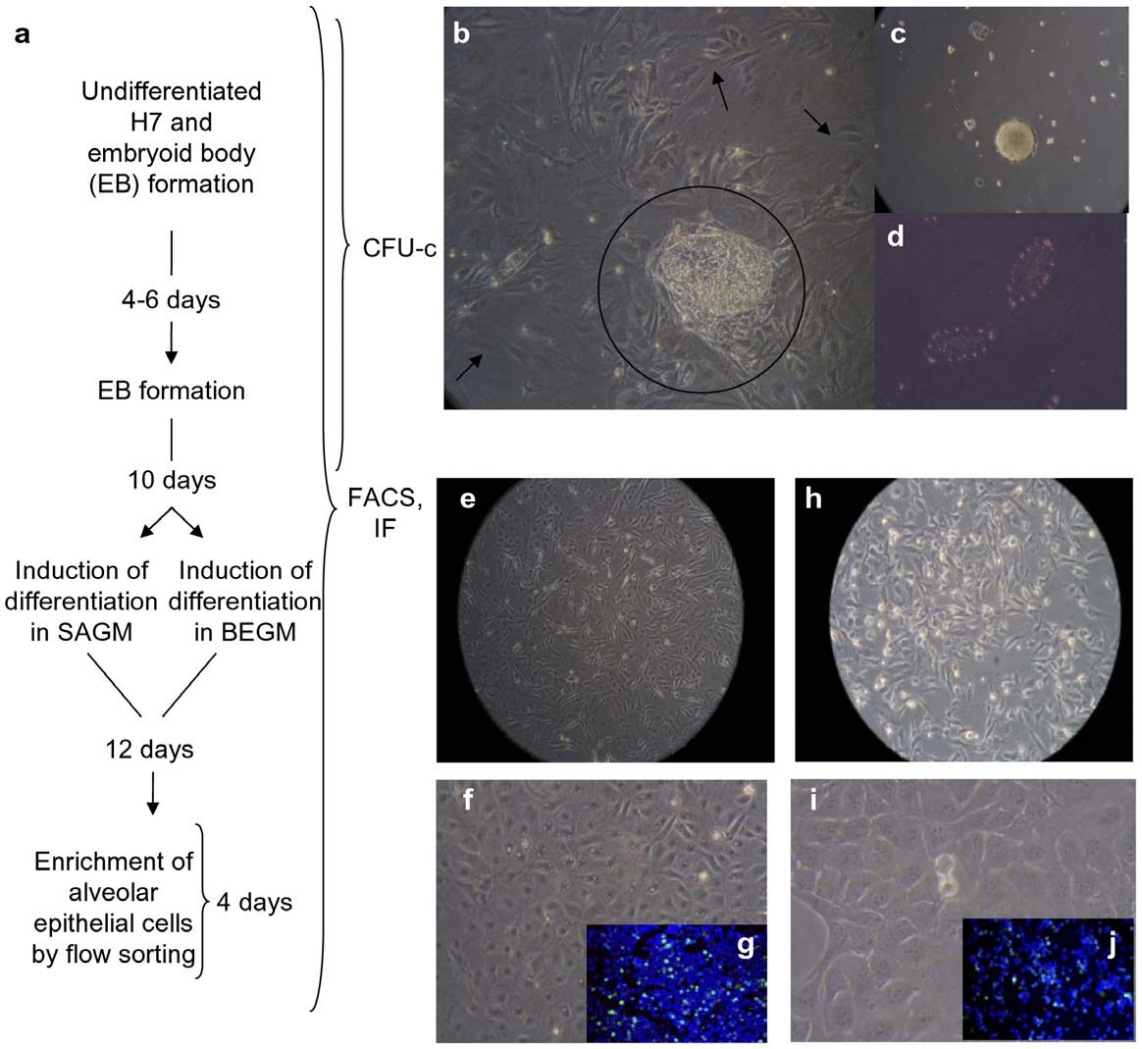
Differentiation of H7 hES cells to lung epithelial cell-specific lineages. **a** Outline of formation of EBs from H7 hES cells and differentiation to alveolar epithelial cells in SAGM and BEGM. **b** Undifferentiated hES cells (within circle) were expanded on γ-irradiated MEF feeders for 4–6 days followed by formation of **c** EBs in suspension culture overnight after aggregation. **d** day 4 EBs were cultured in ultra-low attachment plates for 10 days and then transferred to gelatin-coated plates and cultured with either **e**–**g** SAGM or **h**–**j** BEGM for 12 days (**g** and **j** are insets of SP-C-FITC^+^ cells). AEII cells were flow sorted as surface SP-C^+^ cells and enriched in SAGM for an additional 4 days to amplify cell numbers for transplantation. At each stage, cells were fixed in chamber slides for IF microscopy [green probe is FITC-conjugated lineage (epithelial) marker and blue probe is DAPI-stained nuclei of live cells]. **f**, **g** show enriched AEII cells and **i**, **j** Clara cells. All the steps were performed in three independent experiments, and the results were reproducible.

**Figure 2:**
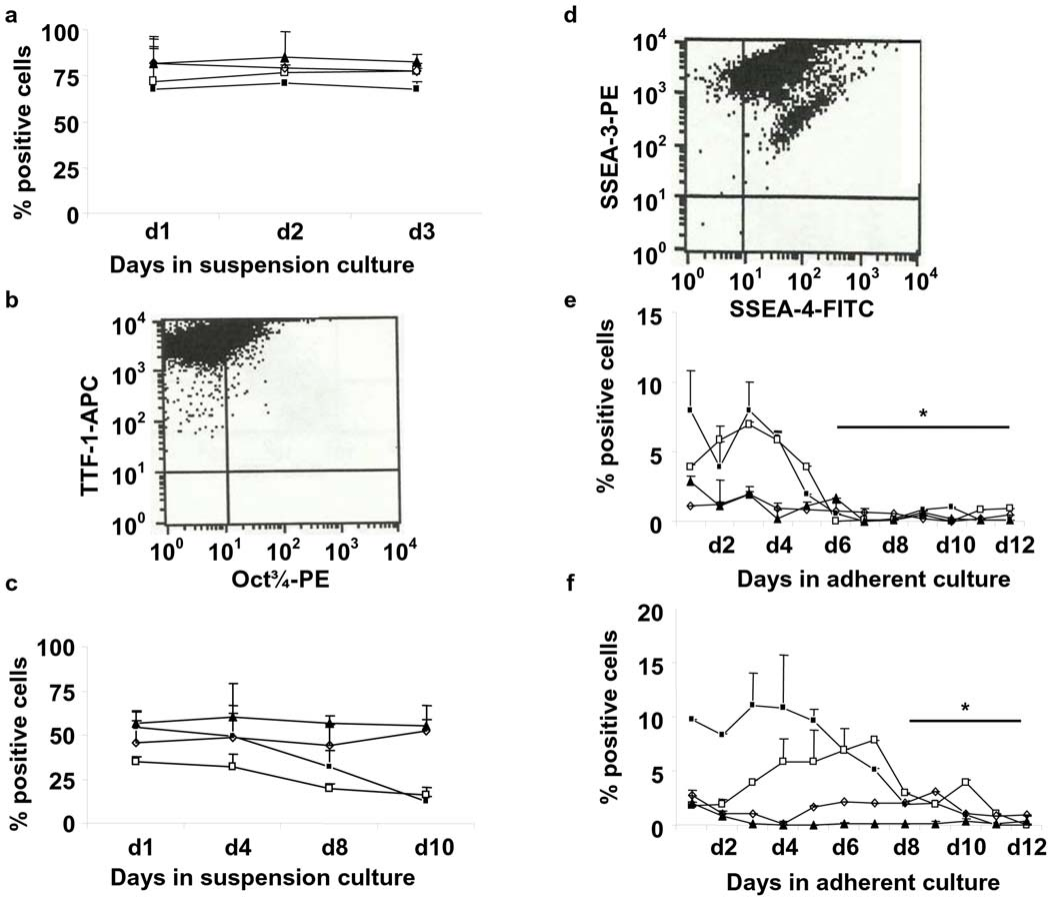
Sequential downregulation of stem cell-specific surface proteins. **a–d** H7 hES cells were cultured in SAGM or BEGM as described in **Figure 1** and made into single cell suspensions and analyzed by FACS. **a** Surface expression of TTF-1, Oct3/4, SSEA-3, and SSEA-4 markers in undifferentiated H7 hES cells over 3 days in γ-irradiated MEF-conditioned media. **b** Representative FACS scattergram for Oct3/4 and TTF-1 of undifferentiated day 1 hES cells is shown with the percentage of cells gated in each quadrant as follows: upper left (UL), 74.27%; upper right (UR), 25.73%; lower left (LL), 0%; and lower right (LR), 0%. **c** EBs grown in suspension culture. **d** Representative FACS scattergram for SSEA-3 and SSEA-4 of day 2 EBs are shown with the percentage of cells gated in each quadrant as follows: UL, 3.5%; UR, 96.47%; LL, 0.02%; and LR, 0.01%. Adherent culture was in either **e** SAGM or **f** BEGM. Symbols: -▪-, TTF-1; □-, Oct3/4; -???-, SSEA-3; -⋄-, SSEA-4 for identification of differentiation stage of the cells in culture. The percentage of positive cells is shown as mean ± SEM (*n* = 3 independent experiments with flow data collected in triplicate). **e**, **f** The asterisk indicates that all values were significant (*P*<0.05) compared to day 0 for **e** days 6–12 and **f** days 8–12 in adherent culture for each of the markers (i.e., TTF-1, Oct3/4, SSEA-3, and SSEA-4). The individual *P*<0.05 values for these marker data points are shown in **Table S2**.

Cells from the hES cell line H7 were differentiated *in vitro* into three lung lineage-specific epithelial cells: AEI cells, AEII cells, and Clara cells as described below. These cells expressed, both intracellularly and on their surface, characteristic marker proteins, detected by fluorescence-activated cell sorting (FACS) and immunofluorescence (IF) microscopy, the mRNA for which were also concomitantly over-expressed as detected by quantitative real-time PCR (qPCR). The protocol for differentiation of pluripotent undifferentiated colonies of H7 hES cells into the lung epithelial cell-specific lineages is shown in **Figure 1a**. After embryoid body (EB) formation (**Figure 1b–d**), EBs were cultured in EB medium for 10 days followed by culture for an additional 12 days in either small airways growth medium (SAGM) (**Figure 1e–g**) or bronchiolar epithelial growth medium (BEGM) (**Figure 1h–j**); BEGM differs from SAGM by the presence of retinoic acid and T3 and absence of BSA. Prior to differentiation, most H7 hES cells expressed high percentages of the early marker of epithelial lung differentiation TTF-1 and pluripotent markers SSEA-3, SSEA-4, and Oct3/4 (**Figure 2a–d**). Following EB formation and differentiation in adherent culture in either SAGM (**Figure 2e**) or BEGM (**Figure 2f**), these markers were downregulated sequentially. Of these markers, Oct3/4 was downregulated first (15.8±4.3% on day 10 in EB medium from 77.9±8.9% positive in the undifferentiated state) followed by TTF-1 (12±2.9% on day 10 from 68±3.8 in the undifferentiated state), whereas SSEA-3 and SSEA-4 expression remained stable (54.9±4.0% and 51.9±14.9% respectively on day 10 in EB medium) (**Figure 2a, c**). During induction of differentiation, all four markers were markedly downregulated to ∼2–8% in either SAGM (**Figure 2e**) or BEGM (**Figure 2f**) within 24 h after transfer to adherent culture and to ∼1% on day 6 in SAGM (**Figure 2e**) and day 11 in BEGM (**Figure 2f**).

### Variation in Growth Media Skews Differentiation of hES Cells to AEI Cell, AEII Cell, and Clara Cell Phenotypes

**Figure 3:**
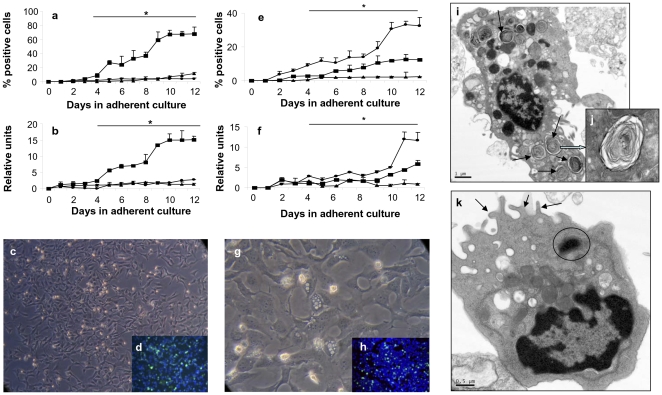
Phenotypic analysis and ultrastructure of hES cells differentiated to lung epithelial cell-specific lineages. EBs derived from H7 hES cells were allowed to form outgrowths and differentiate in EB medium over 10 days and then cells cultured over 12 days in either **a**–**d**, **i**, **j** SAGM or **e**–**h**, **k** BEGM. Expression of intracellular marker proteins was quantitated by **a** FACS analysis of single cell suspensions and **e** IF microscopy with anti-AQP-5, SP-C, and CC-10 monoclonal antibodies used to identify AEI cells, AEII cells, and Clara cells respectively. The mean percentage positive of total cells is shown ± SEM. **b**, **f** qPCR of mRNA of AQP-5, SP-C, and CC-10 normalized to human GAPDH is expressed as relative units ± SEM. Symbols: -???-, AQP-5; -▪-, SP-C; -•-, CC-10. **a**, **b**, **e**, **f** The data are reported as mean ± SEM; *n* = 3 independent experiments with **a** flow data collected in triplicate and **b**, **f** qPCR and **e** IF data collected in duplicate. The asterisk indicates that all values were significant (*P*<0.05) for **a**, **b** SP-C and **e**, **f** CC-10 for days 4–12 in culture when compared against day 0. The individual *P*<0.05 values for days 4–12 in culture for the marker data points are shown in **Table S3**. **c**, **g** H7 hES cells differentiated into epithelial cells viewed at 10× magnification with **d** SP-C-FITC^+^ and **h** CC-10-FITC^+^ cells shown by IF microscopy. **i**, **j** As seen by transmission electron microscopy, representative AEII cells grown in SAGM at day 12 contain characteristic cytoplasmic lamellar bodies (**i**, arrows, scale bar = 1 µm and **j**, scale bar = 200 nm). **k** Clara cell grown in BEGM at day 12 by transmission electron microscopy exhibit apical microvilli on cell surface (arrows) and electron dense secretory vesicles (circle), scale bar = 0.5 µm.

Phenotypic analysis of cells by intracellular and surface marker expression was used to identify lung lineage-specific epithelial cells differentiated from H7 hES cells in either SAGM or BEGM: aquaporin-5 (AQP-5) for AEI cells, SP-C for AEII cells, and Clara cell-specific protein-10 (CC-10) for Clara cells. Cells differentiated in SAGM were predominantly AEII cells (**Figure 3a–d, i, j**). Kinetics of marker expression of the cells over a period of 12 days, identified them as 68.0% AEII cells, 11.7% AEI cells, and 3.9% Clara cells (**Figure 3a**). Representative FACS scattergrams of cells cultured in SAGM are shown in **Figure S1**. mRNA expression of SP-C was increased by 15-fold, AQP-5 by 1.3-fold, and CC-10 by 2.7-fold from the initial differentiation stage (**Figure 3b**). However, when the same culture steps were followed but the induction medium was changed to BEGM (**Figure 3e–h, k**), the hES cells differentiated into 32.6% Clara cells, 12.4% AEII cells, and 2.2% AEI cells with concomitant increase in mRNA expression of CC-10 (6-fold), SP-C (3-fold), and AQP-5 (1.3-fold). By transmission electron microscopy, the predominant cells differentiated in SAGM exhibited the typical morphology of AEII cells (**Figure 3i**) including lamellar bodies (**Figure 3i, j**), whereas those in BEGM had secretory granules characteristic of Clara cells (**Figure 3k**).

### Inhibition of Wnt/β-catenin/CBP Signaling Promotes Differentiaton of hES Cells to AEI Cell Phenotype

**Figure 4:**
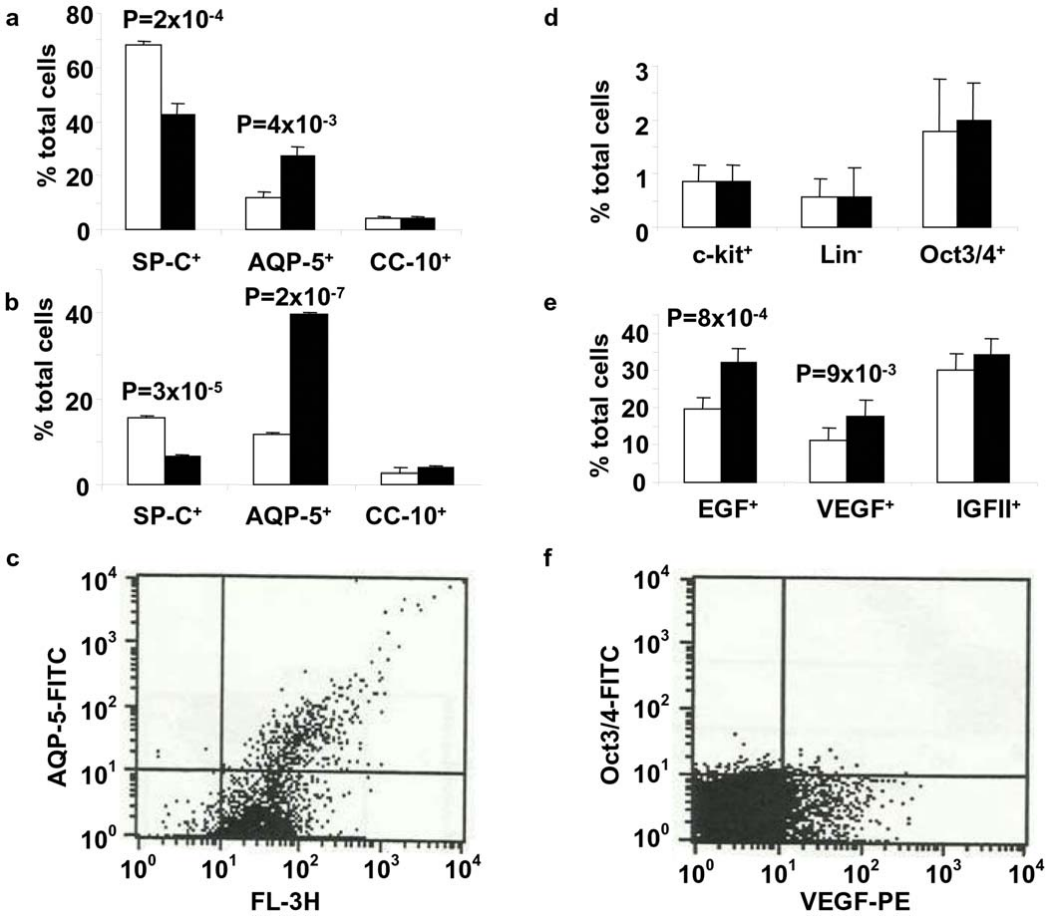
Effect of ICG-001 on differentiation of hES cells in SAGM to AEI cells. Single cell suspensions of H7 hES cells differentiated in SAGM were incubated with 5 µM ICG-001 in culture medium for 12 h and assayed by FACS to identify percentage of AEII cells (SP-C^+^), AEI cells (AQP-5^+^), and Clara cells (CC-10^+^). Percentage of total cells in culture of cells positive for **a** intracellular and **b** surface expression of AEI cell, AEII cell, and Clara cell markers. Surface expression of **d** pluripotent markers for hematopoietic cells (c-kit^+^), lineage-negative (Lin^−^) cells, and Oct3/4^+^ pluripotent cells, and **e** growth factors (EGF^+^, VEGF^+^, IGFII^+^). The percentage of positive cells of total cells in culture is shown as mean ± SEM (*n* = three independent experiments with FACS analyses performed in triplicate). *P*<0.05 values in ICG-001-treated group (open bars) vs. ICG-001-untreated group (solid bars) are shown. Representative FACS scattergrams with the percentage of cells gated in each quadrant shown for expression of **c** AQ-5 (UL, 0.03%; UR, 5.08%; LL, 4.92%; and LR, 89.97%) and **e**: Oct3/4 and VEGF (UL, 1.47%; UR, 0.53%; LL, 86.15%; and LR, 11.85%).

We examined whether the selective β-catenin/CBP inhibitor ICG-001 [22] would modulate the differentiation of undifferentiated hES cells to AEI cells, AEII cells, or Clara cells. As data from independent experiments indicated, incubation of day 12 SAGM-differentiated cells with ICG-001 (5 µM) for 12 h induced differentiation/trans-differentiation of AEII cells to the AEI cell phenotype. Following treatment with ICG-001, there was a significant decrease in AEII cells cultured in either SAGM (**Figures 4** and **S2**) or BEGM (**Figure S3**) as determined by both intracellular (**Figures 4a** and **S3a**) and surface (**Figures 4b** and **S3b**) SP-C^+^ marker expression. In contrast, AEI cells increased significantly in culture in either SAGM or BEGM as determined by intracellular (**Figures 4a** and **S3a**) and surface (**Figures 4b** and **S3b**) AQ-5^+^ expression. The number of Clara cells as assessed by CC-10^+^ expression was unaffected by Wnt- β-catenin inhibitor treatment in either SAGM (**Figure 4a, b**) or BEGM (**Figure S3a, b**). Morphologically, an increase in more flattened cells with larger surface area typical of AEI cells was seen in cells cultured in either SAGM (**Figure S2b, c**) or BEGM (**Figure S3f**) after incubation with ICG-001.

We next examined the effect of modulating Wnt/β-catenin signaling on pluripotent marker expression in hES cells differentiated in SAGM or BEGM: c-kit^+^ (i.e., marker for hematopoietic stem cells as well as early thymocytes, mast cells, melanocytes in skin, and interstitial cells of Cajal), Lin^−^ (i.e., lineage-negative cells that are the traditionally considered as pluripotent cells without expression of lineage markers), and Oct3/4^+^ (expressed universally on pluripotent stem cells). Expression of these pluripotent markers was not significantly affected by modulation of Wnt-β-catenin signaling in cells cultured in either SAGM (**Figure 4d**) or BEGM (**Figure S3c**) except for decreased c-kit^+^ cell number in the cell population cultured in BEGM (**Figure S3c**). The effect of ICG-001 on surface expression of epidermal growth factor (EGF), vascular endothelial growth factor (VEGF), and insulin-like growth factor II (IGFII) was examined by FACS and IF microscopy. Wnt/β-catenin inhibition by ICG-001 induced increased expression of EGF^+^ and VEGF^+^ cells in both SAGM and BEGM, but did not affect the percentage of IGFII^+^ cells (**Figures 4e** and **S3b**).

### Clonogenic Potential of hES Cells Declines with Differentiation

**Figure 5:**
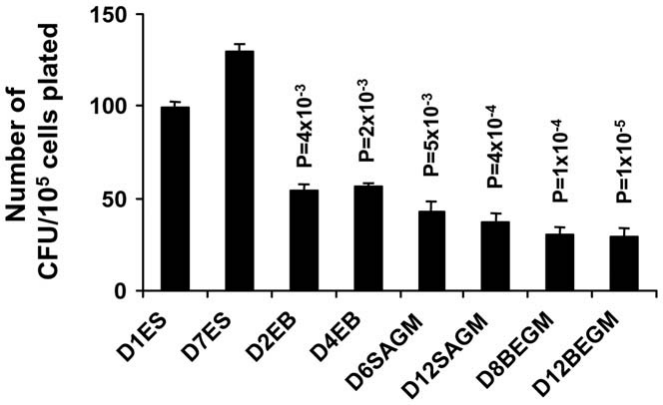
Clonogenic potential progressively declines with increasingly differentiated state of H7 ES cell-derived lung cells. Clonogenic potential was determined in single cell suspensions of undifferentiated H7 ES cells on days 1 and 7 of culture in ES cell medium (i.e., D1ES and D7ES), EBs on day 2 and 4 of culture in EB medium (i.e., D2EB and D4EB), cells from 6 and 12 days of culture in SAGM (i.e., D6SAGM and D12SAGM), and cells from 8 and 12 days in culture in BEGM (i.e., D8BEGM and D12BEGM). Colony forming assays were done with single cell suspensions from each stage in culture and performed in duplicate. Numbers represent colonies counted per 10^5^ cells plated in duplicate after 7 days in culture. The results represent counts from 3 independent experiments as mean ± SEM. *P* value<0.05 was considered significant, compared to colony forming unit (CFU)s from D1ES cells in undifferentiated state.

Lung progenitors are pluripotent cells in the lung. The source of these cells is unclear. We assessed clonogenic potential of hematopoietic progenitor cells by plating cells from either undifferentiated ES or EB samples, or differentiating EB in SAGM and BEGM over different times in culture (**Figure 5**). Viable colonies (i.e., 10^5^ cells were plated from which after growth, colonies with >40 cells were considered for evaluation) increased from 99±3 at day 1 (D1ES, **Figure 5**) to 130±3 on day 7 (D7ES, **Figure 5**) in ES cell medium. As seen in **Figure 5**, a significant decrease in clonogenic potential (as compared to D1ES) was observed in hES cells as they formed EBs (D2EB and D4EB) with the decline progressing as they differentiated in either SAGM (D6SAGM and D12SAGM) or BEGM (D8BEGM and D12BEGM).

### Engraftment of Differentiated hES Cell Transplants in Mice with Lung Fibrosis

H7 hES cells differentiated into lung epithelial cell-specific lineages were transplanted into bleomycin-treated mice to determine whether they would home to injured lung and reduce pulmonary fibrosis. To avoid rejection of the human xenograft cells, sub-lethally irradiated immunocompromised mice (i.e., Rag2γC^−/−^) were used. The differentiated stem cells were delivered by the intratracheal route as we anticipated that the site of injury would facilitate directed migration of the cells towards the fibrotic lesions within the lungs. On day 7 after bleomycin treatment, Rag2γC^−/−^ mice received a single intratracheal instillation of 10^5^ cells from the day 12 culture in SAGM without (i.e., Bleo/hES+SAGM group) or with 5 µM ICG-001 (i.e., Bleo/hES+SAGM+ICG-001 group) treatment. Control groups of saline-treated mice (i.e., Saline group) and bleomycin-treated mice (i.e., Bleo/Saline group) received saline intratracheally on day 7. Cells differentiated in BEGM were not employed in these transplantation studies because culture in this growth medium resulted in differentiation into predominantly Clara cells that are found in terminal bronchioles and thus unlikely to be suitable for treatment of an alveolar destructive disease typified by the mouse bleomycin model of pulmonary fibrosis.

**Figure 6:**
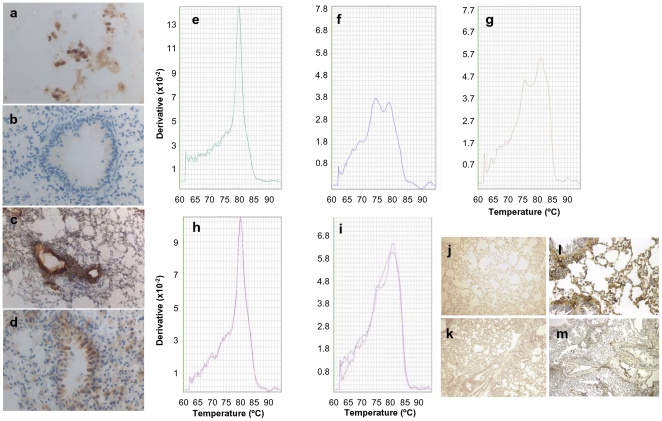
Detection of human cells in lungs of bleomycin-treated mice transplanted with differentiated H7 hES cells. The presence of human cells in the lungs of bleomycin-treated mice transplanted with differentiated H7 hES cells was assessed by **a–d** DAB immunocytochemistry using anti-human nuclear factor antibody on fresh OCT-fixed cryosections, **e–i** qPCR of human Alu sequence, and **j–m** *in situ* hybridization with human pan-centromeric probe. The following groups of Rag2γC^−/−^mice were studied: bleomycin-treated mice administered intratracheally **b**, **g**, **k** (10× magnification) saline (Bleo/Saline group) or 10^5^ H7 hES cells differentiated in SAGM in the **c**, **h**, **l** (60× magnification) absence (SAGM group) or presence of **d**, **i**, **m** (10× magnification) 5 µM ICG-001 (SAGM+ICG-001 group). **a**, **e** positive control of H7 hES cells. **f**, **j** (60× magnification) negative control of mice given only saline intratracheally. **a–d** brown reaction indicate human nuclear-specific antibody staining. 60× magnification. **e–i** qPCR was run using the Alu-specific primer with dissociation curves shown. **l** and **m** brown stains indicate DAB-positive pan-centromeric probe reactions. The data are representative of three independent experiments with *n* = 4 mice per study group in each experiment.

Engraftment of the transplanted human cells in the airways of bleomycin-treated mice was observed in each of the transplant groups. Human cells (from transplants of ES cells differentiated in SAGM alone or SAGM+ICG-001) were detected in mouse airways by immunocytochemistry using anti-human nuclear factor antibody (**Figure 6c**), qPCR of human Alu sequence (**Figure 6h**), and *in situ* hybridization with human-specific pan-centromeric probe (**Figure 6l**). Similarly, hES cells differentiated in SAGM in the presence of ICG-001 were found in the mouse lungs by these methods (**Figure 6d, i, m**). After intratracheal instillation of the hES cells differentiated in SAGM without or with ICG-001 treatment, no human cells were detected in kidney, liver, heart, spleen, bone marrow, or peripheral blood by the qPCR assay of human ALU. Further, analysis of bone marrow, blood, and spleen by FACS with human specific antibodies also failed to detect the presence of human cells indicating that the homing and engraftment of the differentiated hES cells was likely restricted to the airways.

### Differentiated hES Cell Transplants Reduce Pulmonary Inflammation and Fibrosis Induced by Bleomycin

**Figure 7:**
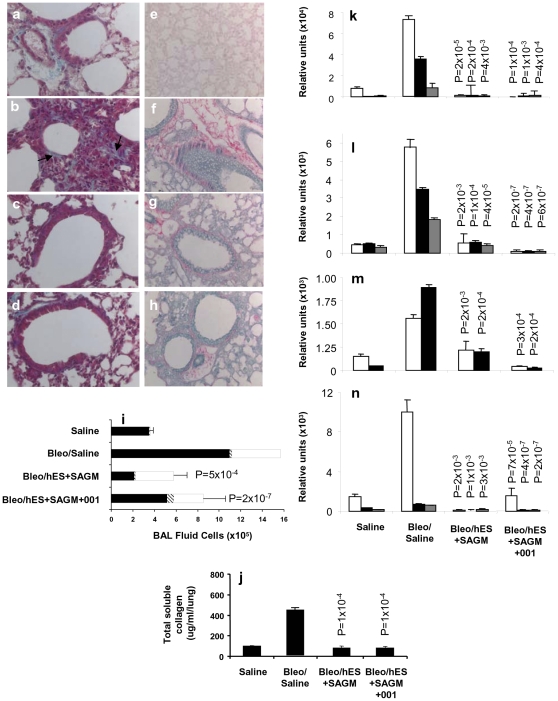
Reversal of lung inflammation and fibrosis by intratracheal transplantation of differentiated hES cells. Transplant groups consisted of **a**, **e**, **i–n** saline-treated control Rag2γC^−/−^ mice (Saline group), **c**, **g**, **i–n** bleomycin-treated Rag2γC^−/−^ mice administered saline (Bleo/Saline group), and bleomycin-treated Rag2γC^−/−^ mice transplanted with 10^5^ H7 hES cells differentiated in SAGM in the either the **c**, **g**, **i–n** absence (Bleo/hES+SAGM group), or **d**, **h**, **i–n** presence of 5 µM ICG-001 (Bleo/hES+SAGM+ICG-001 group) were stained with either **a–d** Masson's trichrome or **e–h** Picro Sirius red staining for collagen. 60× magnification. **i** shows BAL fluid cell counts for macrophages (solid bars), lymphocytes (hatched bars), and neutrophils (open bars) and **j** total soluble collagen/lung measured by the Sircol™ assay. **k–n** show expression by qPCR in relative units of the following genes: **k** collagen genes 1α2, 3α1, and 6α1 [Col 1α2 (white bars), Col 3α1 (black bars), Col 6α1 (gray bars)], **l** [TGFβ_1_ (white bars), TGFβ_2_ (black bars), and TGFβ_3_ (grey bars)], **m** FGF [FGF-1 (white bars) and FGF-2 (black bars)], and **n** VEGF [VEGF-A (white bars), VEGF-B (black bars), and VEGF-C (gray bars)]. *P*<0.05 values compared to bleomycin-treated control group administered saline are shown. Three independent experiments were performed with *n* = 4 mice per study group in each experiment. **i** BAL fluid counts and **k–n** qPCR data were performed in duplicate, and **j** collagen levels were measured in triplicate.

The effect of human stem cells differentiated in SAGM in the absence or presence of treatment with the Wnt/β-catenin inhibitor ICG-001 on bleomycin-induced lung injury was determined (**Figure 7**). Pulmonary pathology in the bleomycin-treated immunocompromised Rag2γC^−/−^ control mice showed a) airway inflammation, as assessed by histology (**Figure 7b**) and increased levels of mononuclear and polymorphonuclear leukocytes in BAL fluid (Bleo/Saline group, **Figure 7i**), and b) alveolar and interstitial fibrosis, as assessed by Masson's trichrome (**Figure 7b**) and Picro Sirius red (**Figure 7f**) staining and increased collagen content (Bleo/Saline group, **Figure 7j**) compared to saline-treated controls (**Figure 7a, e** and Saline group, **Figure 7i, j**).

The bleomycin-induced airway inflammation and fibrosis induced in Rag2γC^−/−^ mice was comparable to that seen in wild-type controls (not shown). Significant reduction of inflammatory cells in BAL fluid and collagen content per lung was observed in the recipient groups receiving hES cells differentiated in either SAGM alone (**Figure 7c, g** and Bleo/hES+SAGM group, **Figure 7i, j**) or in presence of ICG-001 (**Figure 7d, h** and Bleo/hES+SAGM+001 group, **Figure 7i, j**). The increased collagen (i.e., Col1α2, Col3α1, and Col6α1) gene expression found in the lungs of bleomycin-treated control mice (Bleo/Saline group, **Figure 7k**) compared to saline-treated controls (Saline group, **Figure 7k**) was significantly decreased in recipients of H7 hES cells differentiated in SAGM in the absence (Bleo/hES+SAGM group, **Figure 7k**) or presence of ICG-001 treatment (Bleo/hES+SAGM+001 group, **Figure 7k**). Similarly, the increased expression of transforming growth factor (TGF) genes (i.e., TGFβ_1_, TGFβ_2_, and TGFβ_3_; Bleo/Saline group, **Figure 7l**), fibroblast growth factor (FGF) genes (i.e., FGF-1 and FGF-2; Bleo/Saline group, **Figure 7m**), and vascular endothelial growth factor (VEGF) genes (i.e., VEGF-A, VEGF-B, and VEGF-C; Bleo/Saline group, **Figure 7n**) seen in bleomycin-treated mice compared to saline-treated controls (Saline group, **Figure 7l–n**) was reduced by transplantation of the H7 hES cells differentiated in SAGM in the absence (Bleo/hES+SAGM group, **Figure 7l–n**) or presence of ICG-001 (Bleo/hES+SAGM+001 group, **Figure 7l–n**). Teratoma formation was not observed in the lungs or other tissues of the Rag2γC^−/−^ mice transplanted with H7 hES cells differentiated in SAGM in the absence or presence of ICG-001.

### Differentiated hES Cell Transplants Increase Airway Epithelial Cells and Progenitors in Mice with Pulmonary Fibrosis

**Figure 8:**
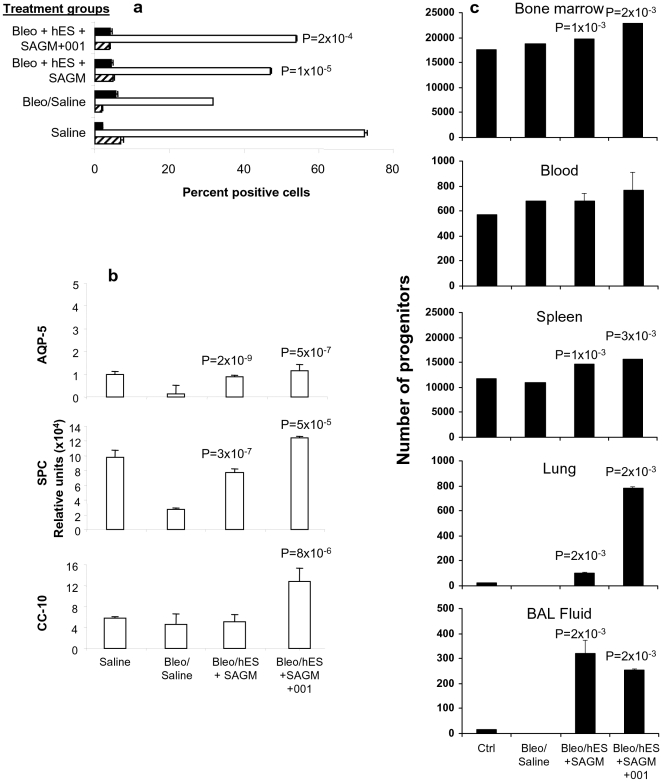
Effect of differentiated H7 hES cells on airway epithelial cells and progenitors in bleomycin-treated mice. Lungs of control (saline-treated) Rag2γC^−/−^ mice (Saline group), bleomycin-treated Rag2γC^−/−^ mice administered saline (Bleo/Saline group), and bleomycin-treated Rag2γC^−/−^ mice transplanted with 10^5^ H7 hES cells differentiated in SAGM in the either the absence (Bleo/hES+SAGM group), or presence of 5 µM ICG-001 (Bleo/hES+SAGM+ICG-001 group) underwent **a** FACS or **b** qPCR analyses for expression of AQP-5, SP-C, and CC-10 markers of AEI cells, AEII cells, and Clara cells respectively. **a** Percent positive cells in lung tissues digested by collagenase type IV treatment for FACS analysis of alveolar epithelial cells ± SEM. *n* = 4 per group. *P value<0.05 compared to d 14 bleomycin-treated non-transplanted mouse lung. Symbols used are: CC-10 (solid bars), AQP-5 (open bars), and SP-C (hatched bars). **b** Lung tissue was homogenized, total RNA extracted, and mRNA expression for AQ-5, SP-C, and CC-10 detected by qPCR as calculated by relative index of Ct values normalized to GAPDH by qPCR and shown as relative units. *P*<0.05 compared to bleomycin-treated non-transplanted lung values are shown. **c** To quantitate hematopoietic progenitors, CFU-C were counted after culture in semi-solid methylcellulose from bone marrow, blood, spleen, lung, and BAL fluid obtained from the study groups. 7 days after culture, the number of bone marrow cell CFU-C was expressed as total number of cells derived per femur, blood CFU-C per 1 ml of plated heparinized whole blood, and spleen CFU-C per spleen. 14 days after culture, lung parenchymal and BAL fluid cell CFU-C were calculated as per single lung. CFU-C was performed in duplicate in three independent experiments with *n* = 4 mice per study group in each experiment; CFU-C are expressed as mean ± SEM with *P*<0.05 compared to the Bleo/Saline group shown.

After enzymatic digestion of lung tissue by collagenase type IV treatment, FACS analysis was performed to quantitate AEII cells (i.e., SP-C^+^), AEI cells (i.e., AQP-5^+^), and Clara cells (i.e., CC-10^+^). AEII cells decreased from 7% at baseline to 2% by day 14 post-bleomycin administration (**Figure 8a**). AEI cells decreased from 72% on day 0 to 53% on day 7 and 31.5% on day 14 post-bleomycin treatment (**Figure 8a**). By day 14, AEI cells increased significantly in the bleomycin-treated groups receiving transplants of H7 hES cells differentiated in SAGM in the absence or presence of treatment with ICG-001. On day 14, the number of Clara cells in the transplant groups was not changed compared to bleomycin-treated controls administered saline instead of human cells on day 7 (**Figure 8a**). Gene expression for marker proteins for AEI cells (i.e., AQP-5^+^), AEII cells (i.e., SP-C^+^), and Clara cells (i.e., CC-10^+^) was also performed (**Figure 8b**). Compared to the bleomycin-treated control group (without transplantation of human cells), expression of AQP-5 and SP-C was increased in both transplanted groups (**Figure 8b**). Increased CC-10 expression was observed only in bleomycin-treated recipients of hES cells differentiated in SAGM and treated with ICG-001 (**Figure 8b**).

Quantitation of colony forming units in culture (CFU-C) in bone marrow, spleen, lung and BAL fluid of bleomycin-treated Rag2γC^−/−^ mice in the transplant groups was assessed (**Figure 8c**). In comparison to bleomycin-treated controls given saline, the number of progenitors significantly increased in the bone marrow, blood, spleen, lung, and BAL fluid of both transplant groups of cells differentiated in SAGM in the absence or presence of ICG-001; lung progenitors were markedly increased in the SAGM + ICG-001 group compared to the SAGM alone transplant group (**Figure 8c**). Progenitor number in the peripheral blood of the SAGM ± ICG-001 transplant groups was not significantly different from the bleomycin-treated control group administered saline (**Figure 8c**). In exclusively hematopoietic tissues [i.e., bone marrow and peripheral blood, we detected all of the following: CFU-E (colony-forming unit-erythroid), BFU-E (burst-forming unit-erythroid), CFU-G (colony-forming unit-granulocyte), CFU-M (colony-forming unit-macrophage), CFU-GM (colony-forming unit-granulocyte/macrophage), and CFU-GEMM (colony-forming unit-granulocyte/erythroid/macrophage/megakaryocyte). In lung tissue and BAL fluid, CFU-M and CFU-GM were detected.

## Discussion

This study describes a novel strategy for differentiating hES cells into endodermal lung lineage-specific cells. Alteration of the differentiation medium strikingly modified the pathway of differentiation of EBs into different cell types. Culture of EBs in a commercially available medium used for maintaining primary culture of mature pulmonary alveolar cells SAGM (excluding tri-iodothyronin and retinoic acid) promoted a predominantly AEII cell phenotype. In contrast, culture in a commercially available BEGM (with tri-iodothyronin and retinoic acid but without BSA) promoted differentiation to predominantly bronchiolar alveolar cell (i.e., Clara cell) phenotype. Lung lineage-specific cell differentiation was achieved in a relatively shorter span of time (22 days) in contrast to other reported lung lineage culture conditions [23]. These culture media, normally used to maintain and grow mature cells, could successfully induce differentiation of pluripotent embryonic stem cells into three types of mature lung lineage-specific non-ciliated cells. Whereas AEI cells and AEII cells are found in the alveolar areas, Clara cells are found in terminal bronchioles. This study demonstrates that from the same clonal population of undifferentiated hES cells, tissue engineering can be used to skew differentiation into one or another type of functionally competent mature cells and that blocking the β-catenin/CBP interaction without interfering with the β-catenin/p300 interaction by the selective Wnt/β-catenin inhibitor ICG-001 induces induction/differentiation from one differentiated cell type into another.

Ultrastructure of the cells by transmission electron microscopy indicated that AEII cells derived from the hES cells had characteristic lamellar bodies in their cytoplasmic vacuoles and Clara cells had characteristic secretory granules. The undifferentiated H7 cells were cultured not on a feeder of irradiated MEFs as is the usual practice but in a MEF-conditioned media that maintained a pure human cell population to be used in transplantation studies to enable detection of the xenograft in recipient mouse tissue without mouse feeder fibroblast contamination. We observed that the percentage of differentiated AE cells was not as high as 68% when the culture time in EB was shortened and may explain why EBs are an essential intermediate step before pluripotent cells may be induced to differentiate in differentiation medium. The sequential downregulation of the pluripotent markers SSEA-3, SSEA-4, and Oct3/4 indicated that the mechanisms controlling the differentiation process follow a hierarchial order in the process of differentiation, and these proteins may play key roles in deciding the fate of the cell. Oct3/4 is a mammalian transcription factor expressed by early embryo cells and germ cells. It is essential for the identity of the pluripotent founder population in the mammalian embryo and required to sustain stem cell self-renewal [24], [25]. Indeed there have been reports that transient over-expression of Oct3/4 leads to massive epithelial hyperplasia that resolves after Oct3/4 expression is switched off, and the expanded pools of tissue-specific progenitor cells differentiate normally [26], [27]. The sequence of downregulation of the pluripotent markers before induction of differentiation may be key in endodermal lineage differentiation (**Figure 3**). SSEA-3 and SSEA-4 are characteristic markers of the H7 cell line that does not express SSEA-1. Both SSEA-3 and SSEA-4 showed a less rapid downregulation while still in the EB medium and percentage positive cells at day 10 are ∼50% of the total population. This may indicate a regulatory mechanism by these proteins that may delay either ectodermal or mesodermal differentiation. TTF-1 (thyroid transcription factor-1) plays an important role in lung development (co-expression of TTF-1 and SP-C and branching morphogenesis in lung. [28], [29]. TTF-1 exhibits a similar rapid downregulation by day 10 in EB medium and may also be a necessary signal for endodermal differentiation to follow.

Detailed characterization of the differentiated cells and the kinetic profile of lineage-specific marker upregulation showed that as the ES cells differentiated into AEII cells as indicated by the markedly increased expression of SP-C marker protein for AEII over time in culture, those for AEI cells (AQP-5^+^) and Clara cells (CC-10^+^), maintained a relatively low expression. It is possible that the cell type found to have been preferentially differentiated may secrete factors suppressing differentiation into another cell type. The decreased clonogenic potential of EBs from their undifferentiated precursors suggests a control mechanism operative during this stage that favors differentiation over proliferation. These results indicate that differentiated cells derived from the hES cells are likely incapable of proliferation on their own once terminally differentiated or their colony forming power may be compromised upon terminal differentiation.

This study demonstrated that ICG-001, a specific inhibitor of the Wnt-β catenin pathway induced differentiation of AEII cells to AEI cells, identified by characteristic marker antigens using FACS, IF, and immunocytochemical staining techniques. By our protocol, pluripotent cells were first differentiated to AEII and AEI cells in SAGM and then ICG-001 likely differentiated some of the differentiated AEII cells to AEI cells by a trans-differentiation process. The Wnt/β-catenin or canonical Wnt signaling pathway is characterized by the nuclear accumulation of β-catenin, where it forms a complex with members of the T cell factor (TCF)/lymphoid enhancer factor-1 (LEF-1) family of transcription factors [reviewed in [30]]. β-catenin subsequently recruits the transcriptional coactivators, Creb-binding protein (CBP) or p300 [31], [32]. Despite the fact that they are highly homologous, recent investigations demonstrate that β-catenin/CBP and β-catenin/p300 transcriptional complexes regulate unique subsets of genes. Further, a switch from β-catenin/CBP to β-catenin/p300-driven transcription is associated with the initiation of differentiation [15], [16], [33]. Wnt/β-catenin signaling promotes self-renewal in a variety of tissue stem cells, including neuronal stem cells and hematopoeitic stem cells [34]. However, activation of the Wnt/β-catenin pathway can either promote or inhibit differentiation depending on the experimental circumstances [35]–[37]. To explain these dichotomous activities of Wnt signaling, recent data indicate that β-catenin/CBP-driven transcription is critical for proliferation without differentiation (e.g., in both normal and cancer stem/progenitor cells); whereas a switch to β-catenin/p300-mediated gene expression is an essential first step in initiating normal cellular differentiation [15], [16], [22], [33]. Since we found recently ICG-001 to reverse fibrosis in a bleomycin-induced lung fibrosis model in mice [21], we examined its effect on hES cell differentiation. Surprisingly, ICG-001 reduced the 68% (SP-C^+^) AEII cells in the heterogeneous cell population in SAGM to 42.5% with a simultaneous increase of the percentage of AQP-5^+^ AEI cells from 12% to 27% by intracellular staining (**Figure 4a**). In BEGM, the AEII population decreased from 19% to 5%, whereas AEI cells increased from 5% to 17% (**Figure S3a**). Of note, the Clara cell percentage remained unaltered by ICG-001 in both SAGM (**Figure 4a**) and BEGM (**Figure S3a**). These results indicate that ICG-001 induces differentiation of AEII cells to AEI cells.

We used a mouse transplantation model of bleomycin-induced lung fibrosis to test whether the lung epithelial cells derived from the hES cells would home to the injured airways and ameliorate pulmonary fibrosis. Rag2γC^−/−^ mice were chosen as recipients as they were found to be ideal candidates for xenograft transplantation and they were further sub-lethally irradiated to decrease the participation of the recipient's own progenitor population either in the lung itself or from bone marrow or from other adult tissues and also to reduce the probability of a graft rejection. Differentiation of the hES cells in SAGM led to an increase in the number of hematopoietic progenitors (i.e. BFU-E, CFU-E, CFU-G, CFU-M, CFU-GM, and CFU-GEMM) in all compartments (i.e., bone marrow, spleen, lung, and BAL fluid) except blood *ex vivo*. We detected CFU in various tissue compartments from the bleomycin-treated mice administered the lung-lineage differentiated hES cell transplants. Specifically, hematopoietic-like colonies were detected in bone marrow and blood and both epithelioid-like and hematopoietic-like colonies were detected in the tissues of the lung and BAL fluid of transplant recipients (**Figure 8**). The fact that they were able to grow colonies in methylcellulose indicates that they are progenitors. Treatment with ICG-001 led to a further marked increase in progenitors in the lungs. Since transplanted mice were not exposed to ICG-001 *in vivo*, this suggests that *in vitro* modification of the cells led to a persistent influence on progenitor number in widespread organs. We hypothesize that homed hES cell-derived lung epithelial cell lineage-specific cells may either influence the local cells to proliferate at a higher rate or by a paracrine effect to modulate the secretion of key growth factors and stromal components that favor a) regeneration of lost functional tissue and b) increased turnover of ECM proteins that are hallmarks of a profibrotic process. This was indicated by RT-PCR analyses showing significant reduction in ECM (i.e., collagen and fibronectin) and profibrotic (i.e., TGFβ, FGF, and VEGF) genes in the lungs of bleomycin-treated mice transplanted with hES cells differentiated in SAGM in either the absence or presence of ICG-001.

Previous engraftment studies in the lungs and other organs have shown that extraneously administered cells have the capacity to home to and expedite the repair process during injury [23], [38]–[42]. Studies with ciliated cells in pulse chase experiments have shown that ciliated cells transiently change their morphology but do not proliferate or trans-differentiate in response to lung injury as part of the repair process [43]. Non-ciliated pulmonary lineage-specific cells however, show both proliferation and trans-differentiation but not alteration in morphology. Our study corroborates this recent finding. Since this was a short-term engraftment experiment and the cells had only a week to home and engraft, true engraftment into lung tissue may not have occurred. Alternatively, the differentiated human cells homing to the foci of injury and fibrosis, mainly in the smaller airways may have fused with local cells there. This homing of cells to the foci of injury in the small airways was associated with down-modulation of the composite profibrotic phenotype in the injured lung. No teratoma formation in the transplanted tissue was observed.

In summary, using a xenograft transplantation model, we have demonstrated that differentiated lung cells derived from hES cells can reverse fibrosis by homing to airways. The engrafted cells may reduce fibrosis either by directly replacing fibrotic tissue or indirectly by paracrine secretion of factors that reduce deposition of collagen and other ECM components. This engraftment was also associated with increased levels of AEI and AEII cells and progenitors in the lungs. The data suggest that there are circulating progenitors of both hematopoietic and non-hematopoietic origin that have been mobilized from their respective niches in bone marrow or adult tissue (e.g., splenic lymphoid tissue or the lung) that home to the injured lung via the systemic and pulmonary circulation. These studies are an important step toward development of cell-based therapy to potentially replenish damaged tissue in acute or chronic lung injury.

## Materials and Methods

### Ethics Statement

All animal work was conducted according to relevant national and international guidelines as approved by the University of Washington Institutional Animal Care and Use Committee (IACUC) under Protocol Number 2164-04.

### Expansion of H7 hES Cells

NIH approved (NIH code WA07) undifferentiated hES cell line H7 was obtained from WiCell Research Institute (Madison, WI) [44], and cells from passage 25 to 35 were used. For propagation of the H7 cells in undifferentiated state, the ES cells were initially grown on primary mouse embryonic fibroblast (MEF) feeder cells prepared from timed pregnant CF-1 female mice (day 13.5 of gestation) that had been γ-irradiated with 3000 rads for 5 min, and then directly in conditioned medium in which the above γ-irradiated MEF cells were cultured to ensure purity of human cells and progressively eliminate any mouse feeders from the cultures. The medium contained Dulbecco's Modified Essential Medium (DMEM), 10% heat-inactivated fetal bovine serum (FBS), and 2 mM L-glutamine as described previously [45]. The hES cells were cultured in ES medium [i.e., knockout (KO) DMEM supplemented with 20% knock-out serum replacement (KOSR; Invitrogen, Carlsbad, CA), 1 mM sodium pyruvate, 0.1 mM 2β-mercaptoethanol (ME) (Sigma-Aldrich Corporation, St Louis, MO), 0.1 mM minimum essential media (MEM), 1% nonessential amino acids (NEAA; Mediatech, Herndon,VA), 1 mM L-glutamine, and 2 ng/ml basic fibroblast growth factor (bFGF) (R&D Systems, Minneapolis, MN)]. For cell culture, 6-well 10 cm^2^ tissue culture plates, coated with 0.1% gelatin were used, and all cultures were done in a humidified 5% CO_2_ incubator at 37°C. The protocol for induction of alveolar epithelial differentiation of hES cells was adapted from established methods [17], [19], [46], as shown in **Figure 1a**.

### Embryoid Body Formation

On the day of passage, hES cell colonies were inspected, and only hES cell cultures containing colonies with well-defined boundaries and minimum differentiation were used. Undifferentiated hES cells were treated with 1.2 U/ml dispase (Invitrogen) dissolved in Ca^2+^- and Mg^2+^-free phosphate-buffered saline (PBS; Mediatech) supplemented with 10% ES cell-qualified fetal bovine serum (FBS; Invitrogen) at 37°C until the hES cell colonies nearly detached from the plates. Colonies were then washed off the plates, washed twice in ES cell medium without bFGF, and resuspended in EB medium [i.e., KO DMEM, 20% KOSR, 20% non-heat-inactivated fetal calf serum, 1% NEAA, 1 mM L-glutamine, and 0.1 mM 2β-ME]. Cells were transferred to Corning 6-well ultra-low attachment plates (Corning Inc. Lifesciences, Lowell, MA) and grown for 4 days in suspension culture in ultra-low attachment plates.

### Generation of Non-ciliated Pulmonary Epithelial Cells

Two different culture media were employed to generate non-ciliated pulmonary epithelial cells. EBs were transferred to adherent culture in 0.1% gelatin-coated tissue culture plates by limited dispase digestion. One group of EBs was cultured for 12 days in small airways growth medium (SAGM) [i.e., Clonetics small airways basal medium (Cambrex Bioscience, Walkersville, MD), bovine pituitary extract 30 µg/ml, insulin 5 µg/ml, hydrocortisone 0.5 µg/ml, gentamycin sulfate-amphotericin B 0.5 µg/ml, bovine serum albumin 0.5 mg/ml, transferrin 10 µg/ml, epinephrine 0.5 µg/ml, and recombinant human epidermal growth factor (rh EGF) 0.5 ng/ml] refreshing media every other day. Retinoic acid 0.1 ng/ml and triiodothyronine (6.5 ng/ml) were excluded from SABM following Ali et al. [17]. From the day 12 culture in SAGM, alveolar epithelial cells were flow sorted based on surface expression of SP-C and AQP-5. The >90% SP-C^+^ and AQP-5^+^ flow-sorted cells were grown in the SAGM medium for 4 more days.

A second group consisted of EBs cultured in bronchiolar epithelial growth medium (BEGM) [i.e., Clonetics bronchiolar epithelial basal medium (Cambrex Bioscience), bovine pituitary extract 30 µg/ml, insulin 5 µg/ml, hydrocortisone 0.5 µg/ml, gentamycin sulfate-amphotericin B 0.5 µg/ml, retinoic acid 0.1 ng/ml, transferrin 10 µg/ml, triiodothyronine 6.5 ng/ml, epinephrine 0.5 µg/ml, and rh EGF 0.5 ng/ml] and fed similarly every other day with fresh medium.

### Phenotypic Analysis of Cells

Immunostaining was performed using specific antibodies conjugated to various fluorochromes such as fluorescein isothiocyanate (FITC), phycoerythrin (PE), allophycocyanin (APC), peridinin chlorophyll protein (PerCP-Cy5.5), and CyChrome (PE-Cy5 and PE-Cy7). The following BD Biosciences Pharmingen (San Diego, CA) antibodies were used for cell surface staining: APC-conjugated CD45 (30F-11), FITC-conjugated CD3 (145-2C11), PE-Cy5-conjugated B220 (RA3-6B2), APC-conjugated GR-1 (RB6-8C5), PE-conjugated Mac1 (M1/70), FITC-conjugated Sca-1, and PE-Cy7-conjugated CD117 (c-kit). PE-Cy5-conjugated F4/80 [Cl: A3-1 (F4/80)] was obtained from Serotec Ltd., Oxford, UK. Purified antibodies (clone number/catalog number/antibody type/concentration) to the following mouse antigens were obtained from Santa Cruz Biotechnology (Santa Cruz, CA): SSEA-3 (631/sc-21703/rat monoclonal IgM/200 µg/ml), SSEA-4 (813-70/sc-21704/mouse monoclonal IgG_3_/200 µg/ml), Oct-3/4 (H-134/sc-7705/goat polyclonal IgG/200 µg/ml), SP-C (C-19/sc-7705/goat polyclonal IgG/200 µg/ml), SP-D (245-01/sc-59695/mouse monoclonal IgG_1_/100 µg/ml), AQP-1 (L-19/sc-9878/goat polyclonal IgG/200 µg/ml), AQP-5 (G-19/sc-9890/goat polyclonal IgG/200 µg/ml), CC-10 (S-20/sc-9773/goat polyclonal IgG/200 µg/ml), EGF (C-20/sc-1341/goat polyclonal IgG/200 µg/ml, VEGF (P-20/sc-1836/goat polyclonal IgG/200 µg/ml), TTF-1 (G-17/sc-12524/goat polyclonal IgG/200 µg/ml), CD31 [i.e., platelet endothelial cell adhesion molecule (PECAM-1); V-16/sc-31045/goat polyclonal IgG/200 µg/ml], and goat anti-mouse IgG_3_-FITC (sc-2081/pre-adsorbed, affinity-purified secondary antibody raised in goat against mouse IgG_3_ and conjugated to FITC/400 µg/ml). Irrelevant isotype-matched antibodies were used as controls. FITC-conjugated donkey anti-goat or goat anti-rabbit secondary antibodies were used following incubation with the primary antibodies. *In situ* immunostaining with specific FITC- or PE-conjugated antibodies (and DAPI counterstaining the nuclei of the cells) or ABC staining (DAKO) was done following the manufacturer's protocol. 10^6^ cells were taken per sample in 50 µl cell suspension in ice cold PBS (1×); 10^5^ events were recorded per sort.

For simultaneous surface and intracellular staining, cell-surface antigens were stained as follows: 1 µl conjugated antibody/10^6^ cells in suspension culture for 30 min on ice. After thorough washing, cells were fixed in 4% paraformaldehyde in PBS by vortexing, and incubated at room temperature (RT) for 20 min followed by permeabilization in either 0.1% Tween-20 or 0.25% Triton-X. Intracellular staining was performed with readouts made on a FACScalibur. Different conjugates with widely separated excitation spectral range were used for separating the surface vs. intracellular probes (e.g., PE vs. FITC, FITC vs. APC, APC vs. PE, or APC vs. Cyc-PE).

Cell suspension of 10^6^ cells per microfuge tube was prepared per sample and staining was done by a single step with a master mix of fluorochrome-conjugated monoclonal antibodies or in some cases where the primary antibody was not available in a directly fluorochrome-conjugated form, in two steps of primary unlabeled antibody followed by cross reactive fluorochrome-conjugated specific secondary antibody at 4°C for 30 min followed by rigorous washing (twice) with ice cold PBS. The stained cell preparation was finally resuspended in 50 µl PBS (with 1% bovine serum albumin) and read by FACSCalibur (BD Immunocytometry Systems, San Jose, CA) by using the CELLQuest program. Cells were viewed at first keeping at Side Scatter (SSC; X-axis) and Forward Scatter (FSC; Y-axis) and dead cells gated out by annexin V staining. CD45^−^ cells were then gated out to preempt any blood cells in the lungs, and 10^5^ events were recorded per sample. The unstained axis was FL-3H. In undifferentiated H7 cells, single staining with each antibody was done for TTF-1, Oct3/4, SSEA-3, and SSEA-4. Data from three independent experiments with each sample sorted in triplicate were pooled, and mean ± SEM reported.

### Cell Viability

Viable cells were measured by propidium iodide exclusion using flow cytometry and trypan blue dye exclusion by light microscopy.

### Clonogenic Growth of Cells Derived from hES Cells

To quantitate committed progenitors, CFU-C assays were performed using methylcellulose semisolid media (Stemgenix, Amherst, NY) supplemented with an additional 50 ng of stem cell factor per ml (Peprotech, Rocky Hill, NJ) to promote growth of hematopoietic progenitors. Next, 0.01×10^6^ cells from lung were plated on duplicate 35-mm culture dishes and incubated at 37°C in a 5% CO_2_-95% air mixture in a humidified chamber for 7 days. Colonies generated by that time were counted using a dissecting microscope, and all colony types (i.e., BFU-E, CFU-E, CFU-G, CFU-GEMM, CFU-GM, and CFU-M) were pooled and reported as total CFU-C. Aliquots of 1–10×10^4^ cells were plated per 1 ml of semisolid methylcellulose (CFU-lite with Epo, Miltenyi Biotech, or complete human methycellulose medium, Stem Cell Technologies, Vancouver, BC, Canada). CFU-C frequency was scored morphologically after 10 to 14 days in culture at 37°C, 5% CO_2_, in a humidified incubator.

### Mouse Model of Pulmonary Fibrosis and Transplantation of Differentiated H7 hES Cells

Rag2γC double KO mice (Raγ2γC^−/−^) from Taconic (Hudson, NY) [47] were housed under specific pathogen-free conditions. A single intratracheal dose of 0.075 U/ml of bleomycin in 40 µl saline was administered (day 0). On day 7 after bleomycin treatment, mice were irradiated sub-lethally (300R) prior to transplant to minimize the possibility of graft rejection and then transplanted with 10^5^ differentiated hES cells intratracheally in a 50 µl volume. Transplant groups consisted of cells from day 12 in culture with SAGM alone (Bleo/hES+SAGM group) or with treatment of 5 µM ICG-001 for 6 h at 37°C (Bleo/hES+SAGM+ICG-001 group). Before transplantation, cells were incubated for 30 min at 37°C with a pro-survival cocktail composed of 10 mM ZVAD-FMK (Promega, Madison, WI), 50 nM Bcl-XL BH4 (Transduction Laboratories, Lexington, KY), 0.2 µM cyclosporine A (Sigma-Aldrich Corporation), 100 ng/ml recombinant mouse insulin-like growth factor-1 (IGF-1, Santa Cruz Biotechnologies), and 50 µM of the K_ATP_ channel opener pinacidil (Sigma-Aldrich Corporation) [48], [49]. The cells were heat-shocked for 30 min at 42°C in a water bath followed by return to 37°C before transplantation. The pro-survival cocktail was not included in the injectate. Control groups consisted of mice treated intratracheally (50 µl) with either saline (Saline group) or bleomycin (Bleo/Saline group) on day 0 and saline on day 7.

Mice were sacrificed on day 14 with the total number of cells and CFU-C in bone marrow, spleen, blood, lungs, and bronchoalveolar lavage (BAL) fluid determined. Both femurs were flushed to obtain bone marrow. After intraorbital bleeding, blood counts were extrapolated to a total volume of 2 ml (i.e., total blood volume in 20 gm mouse). Parenchymal cells of a single lung were obtained by enzymatic digestion with 0.1% collagenase type IV for 60 min at 37°C. Cell subsets in BAL fluid (i.e., macrophages, lymphocytes, and neutrophils as a percentage of total leukocytes) were quantitated by monoclonal antibodies conjugated with fluorochromes gated as CD45^+^ cells in FACScan. Analysis of single cell suspensions of cell populations was done by enzymatic digestion with 0.1% collagenase type IV for 60 min at 37°C for detachment from lung tissue, followed by cell counting in Coulter counter and cell subset identification and quantitation by flow cytometry. Lung tissue was extracted for total RNA for qPCR and sections obtained for histochemistry/immunocytochemistry.

### Analysis of Colony Forming Units in Tissue Compartments

The number of bone marrow CFU-C counted 7 days after culture in semi-solid methylcellulose supplemented with 50 ng/ml stem cell factor was calculated as per single femur. Typically 50,000 cells were plated per ml. The number of blood CFU-C grown in semi-solid methylcellulose medium for 7 days was determined per ml of plated heparinized whole blood. The number of splenic CFU-C grown after 7 days in methylcellulose with 500,000 splenocytes plated was extrapolated to total number of splenocytes in one spleen. The number of CFU-C grown after 14 days of culture in semi-solid methylcellulose medium plated with 1×10^6^ cells from dispase-digested mouse lung was calculated on total number of cells obtained from a single lung. The number of CFU-C grown after 14 days of culture in semi-solid methylcellulose medium plated with 1×10^6^ BAL fluid cells was calculated on the total number of cells obtained from BAL fluid from a single lung. The cells were derived from Rag2γC double knockout mice that were sub-lethally irradiated prior to xenograft transplantation with human H7 cells. Thus, progenitor numbers were lower than expected because a) the recipients were severely immunocompromised, b) they were irradiated already depleting progenitor reservoir in their tissues, and c) the 1 week period of homing and engraftment allowed in the experiment was very short to reduce the possibility of teratoma formation. Cells were counted on a Leica DMIL inverted microscope (10×) and photographed with a Canon Power Shot S50 digital camera; at least 40-cell colonies were considered.

### RNA Isolation

Total RNA was extracted from cultured cells (<500/sample) by PicoPure RNA isolation kit (Arcturus, Mountain View, CA). For isolation of total lung RNA, 600 µl of lysing buffer was added to disrupted lung tissue in a 1.5-ml microfuge tube, and lysate was loaded onto a QIAshredder column and centrifuged for 2 min at 13,000 rpm. The homogenized lysate was then mixed with 600 µl of 70% ethanol and applied to an RNeasy mini spin (QIAGEN Inc, Valencia, Calif) column for centrifugation for 15 sec at 13,000 rpm. Next, 700 µl of buffer RW1 and buffer RPE was added and centrifuged sequentially for washing twice. Then, 60 µl of ribonuclease-free water was used to elute total RNA from the RNeasy mini spin column. All total RNA used in the experiments was pure as determined by the ratio of absorbance (A) at 260 vs. 280 nm (A260/A280 ratio >1.9) and stored at −80°C.

### qPCR Analysis

cDNA was made using Superscript III system from Invitrogen and qPCR performed. For qPCR performed in duplicate tubes, the PCR reaction solution contained 0.5 µg of total RNA, 6 mM magnesium chloride, and 0.5 µM of each primer (primer oligonucleotide sequences shown are in **Table S1** in the online supporting information. Other components in the reverse transcriptase PCR master mix included buffer, enzyme, SYBR Green I, and deoxyribonucleotide triphosphate. For reverse transcription, the 20 µl of reaction capillaries were incubated at 50°C for 2 min followed by denaturation at 95°C for 10 min. PCR by initial denaturation at 95°C for 15 sec was followed by annealing at 60°C for 1 min, repeated 45 cycles. Finally, a melting curve analysis was performed by following the final cycle with incubation at 95°C for 15 sec, 60°C for 15 sec, and 95°C for 15 sec. Negative control samples for the qPCR analysis that contained all reaction components except RNA, were performed simultaneously to determine when the nonspecific exponential amplification cycle number was reached. Primers were synthesized by the University of Washington Biochemistry services using Primer Express software. qPCR was performed by the comparative Ct method with SYBR Green PCR core reagents (Applied Biosystems, Foster City, CA) and analyzed using Applied Biosystems 7900HT Real-Time PCR System software SDS 2.2.1.

### Analysis of Collagen Content in Lung

Masson's trichrome and Sirius red stains were used to detect collagen deposition in the lungs [50]. Total amount soluble collagen in the lung was determined as the mean of triplicate tubes for each sample by the Sircol™ quantitative dye-binding collagen assay (Biocolor Ltd., Newtownabbey, Northern Ireland, UK) [50].

### Detection of Human Cells in Mouse Lung

Three methods were employed to detect engrafted derived cells in mouse lung: 1) detection of Alu sequence in transplanted mouse lung RNA was performed by qPCR, using the following primers: GTCAGGAGATCGAGACCATCCC (forward sequence) and TCCTGCCTCAGCCTCCCAAG (reverse sequence), Alu elements are specific to the human genome and are present at ∼1 million copies/diploid sequence, making them a sensitive indicator of human cell content. 2) Immunocytochemistry was performed using a mouse anti-human nuclei IgG_1_ monoclonal antibody (clone 235-1, catalog number: MAB1281; Millipore Corporation, Billerica, MA) that stains nuclei of all human cell types giving a diffuse nuclear pattern with no reactivity against mouse in immunohistochemistry. 5 µm thick sections of 2% paraformaldehyde-fixed OCT-embedded frozen lung tissue were blocked with goat serum at RT for 1 h followed by incubation with the anti-human nuclei antibody overnight at 4°C. After washing in PBS and incubation with secondary goat anti-mouse antibody for 1 h at RT, ABC staining (Vector Laboratories Inc.) was performed following the manufacturer's protocol; and 3) *in situ* hybridization in mouse transplanted lung sections with human-specific pan-centromeric probe was performed in 8 µm thick, methyl carnoy-fixed paraffin-embedded lung sections following the protocol described previously [48].

### Immunohistochemistry

For immunohistochemistry with non-conjugated antibodies, paraffin-embedded lung tissue was deparaffined in xylene, and rehydrated in 100% and 95% ethyl alcohol. Endogenous peroxidase was quenched in methanol with 0.3%–3% hydrogen peroxide for 30 min at RT. Blocking was done for 1 h at RT in PBS containing Ca^2+^ and Mg^2+^ with 1.5% non-immune serum of the species in which the secondary antibody was made. The primary antibody was incubated for 1 h at RT followed by 3 washes in PBS at RT. The secondary antibody was applied and incubated. ABC staining (Vector Laboratories Inc, Burlingame, CA) was performed following the manufacturer's protocol.

### Immunofluorescence Microscopy

Photographs were taken with a Leica DMIL inverted microscope (Leica Microsystems GmbH, Wetzlar, Germany) and a Zeiss ApoTome (Carl Zeiss Microimaging GmbH, Göttingen, Germany). IF photographs were taken with a Zeiss Axiovert 200 M microscope and Axiocam MRm and merged using Axiovision 4.6 software.

### Transmission electron microscopy

For transmission electron microscopy, the cells were fixed with warm ½ Karnovsky's fixative (1∶1 with buffer) after removal of the culture medium and washed with 0.1 M cacodylate buffer for 10 min. After the fixative was removed, the sections were incubated in pure fixative for 30–60 min. The cells were gently scraped using a standard Sarstedt cell scraper, and placed into Eppendorf tubes and spun down at 1500 rpm for 5 min. After addition of new fixative, the cells were resuspended and stored at 4°C overnight. After 3 washes for 5 min in 0.1 M cacodylate buffer, cells were centrifuged and 1% osmium tetroxide in 0.1 M cacodylate buffer added and incubated for 1–2 h at 4°C followed by 3 washes in 0.1 M cacodylate buffer for 5 min. Dehydration was done in graded series of ethyl alcohol (i.e., 50%, 70%, 95%, 2×100%) for 15 min and two washes in propylene oxide for 15 min. Embedding was done in 1∶1 propylene oxide/Epon resin overnight with Eppendorf tubes capped. The next day, cells were centrifuged and fresh 100% Epon resin added for 2–4 h. Polymerization was done in a 60°C oven overnight in Eppendorf tubes. 70–100 nm thick sections were made on a copper grid using a Leica EM UC6 ultramicrotome (Leica Microsystems GmbH, Wetzlar, Germany). Sections were viewed in a JEOL JEM-1230 transmission electron microscope (JEOL Ltd., Tokyo, Japan), equipped with an Ultrascan 1000™ 2k×2k CCD camera (Gatan, Inc., Pleasanton, CA), and photomicrographs taken using Gatan Digital Microscope software.

### Statistical Analysis

The data are reported as mean ± SEM. Statistical differences among samples were tested by Student's *t* test. *P* value<0.05 was considered statistically significant.

## Supporting Information

Figure S1 **FACS scattergrams of hES cells differentiated to lung epithelial cell-specific lineages.** EBs were differentiated in EB medium over 10 days and then cells cultured over 12 days in SAGM as described in **Figure 3**. FL-3H denotes gating around cells negative for all pluripotent markers. **a** day 1, **b**, **c** day 8, and **d** day 12 scattergrams are shown. Cells double positive for SP-C and non-pluripotent markers were considered as lung lineage-specific differentiated cells consistent with an AEII phenotype. The data shown are representative of *n* = 3 independent experiments. The percentage of cells gated in each quadrant is shown for expression of **a** SP-C (UL, 3.58%; UR, 0.67%; LL, 88.72%; and LR, 7.03%), **b**: SP-C (UL, 0.58%; UR, 0.47%; LL, 58.38%; and LR, 40.57%), **c**: SP-C and CC-10 (UL, 3.33%; UR, 3.70%; LL, 58.77%; and LR, 34.20%), and **d**: SP-C and TTF-1 (UL, 0.01%; UR, 32.42%; LL, 0.12%; and LR, 67.45%).
(TIF)

Figure S2 **Effect of ICG-001 on cell morphology of hES cells in SAGM.** H7 hES cells differentiated in SAGM were incubated with 5 µM ICG-001 in culture medium for 12 h as described in **Figure 4**, and cell morphology was assessed **a** before, and **b**, **c** after incubation with ICG-001. **a**, **b** 10× and **c** 40× magnification. The data shown are representative of *n* = 3 independent experiments.
(TIF)

Figure S3 **Effect of ICG-001 on hES cells differentiated in BEGM.** Single cell suspensions of human H7 hES cells differentiated in BEGM and incubated in the **a–d** (open bars), **e** absence or **a–d** (solid bars), **f** presence of 5 µM ICG-001 in culture medium for 12 h underwent FACS to identify percentage by **a** intracellular and **b** surface expression of surface markers for AEII cells (SP-C^+^), AEI cells (AQP-5^+^), and Clara cells (CC-10^+^) and expression of **c** pluripotent markers (c-kit^+^, Lin^−^, Oct3/4^+^) and **d** growth factors (EGF^+^, VEGF^+^, IGFII^+^). The percent positive cells of total cells in culture are shown as mean ± SEM (triplicate sampling from three independent experiments). *P*<0.05 values in ICG-001-treated group vs. untreated group are shown. **e**, **f** 40× magnification.
(TIF)

Table S1 **Mouse primers for qPCR.**
(DOCX)

Table S2 **P<0.05 for** **Figure 2** **:** **“Sequential downregulation of stem cell-specific surface proteins” panels e and f.** *Figure 2e* *data points where P<0.05.* *Figure 2f* *data points where P<0.05.*
(DOCX)

Table S3 **P<0.05 for** **Figure 3** **:** **“Phenotypic analysis and ultrastructure of hES cells differentiated to lung epithelial cell-specific lineages” panels a, b, e, and f.** *Figure 3a* *marker data points where P<0.05.* *Figure 3b* *marker data points where P<0.05.* *Figure 3e* *marker data points where P<0.05.* *Figure 3f* *marker data points where P<0.05.* NS, Not Significant.
(DOCX)
