# Supplementary figures and images for: Human Embryonic Stem Cells Differentiated to Lung Lineage-Specific Cells Ameliorate Pulmonary Fibrosis in a Xenograft Transplant Mouse Model
Source: PLoS One. 2012 Mar 28;7(3):e33165. doi: 10.1371/journal.pone.0033165 (PMC3314647; doi:10.1371/journal.pone.0033165)

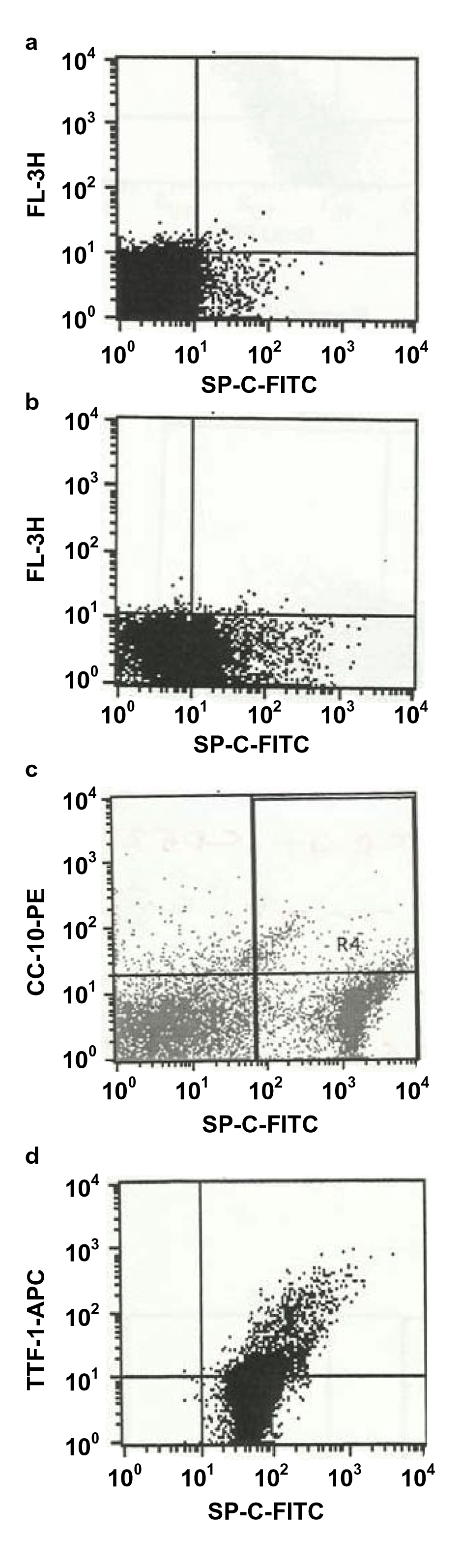

Supplement: Figure S1 — FACS scattergrams of hES cells differentiated to lung epithelial cell-specific lineages. EBs were differentiated in EB medium over 10 days and then cells cultured over 12 days in SAGM as described in Figure 3 . FL-3H denotes gating around cells negative for all pluripotent markers. a day 1, b, c day 8, and d day 12 scattergrams are shown. Cells double positive for SP-C and non-pluripotent markers were considered as lung lineage-specific differentiated cells consistent with an AEII phenotype. The data shown are representative of n = 3 independent experiments. The percentage of cells gated in each quadrant is shown for expression of a SP-C (UL, 3.58%; UR, 0.67%; LL, 88.72%; and LR, 7.03%), b: SP-C (UL, 0.58%; UR, 0.47%; LL, 58.38%; and LR, 40.57%), c: SP-C and CC-10 (UL, 3.33%; UR, 3.70%; LL, 58.77%; and LR, 34.20%), and d: SP-C and TTF-1 (UL, 0.01%; UR, 32.42%; LL, 0.12%; and LR, 67.45%). (TIF) [file pone.0033165.s001.tif]

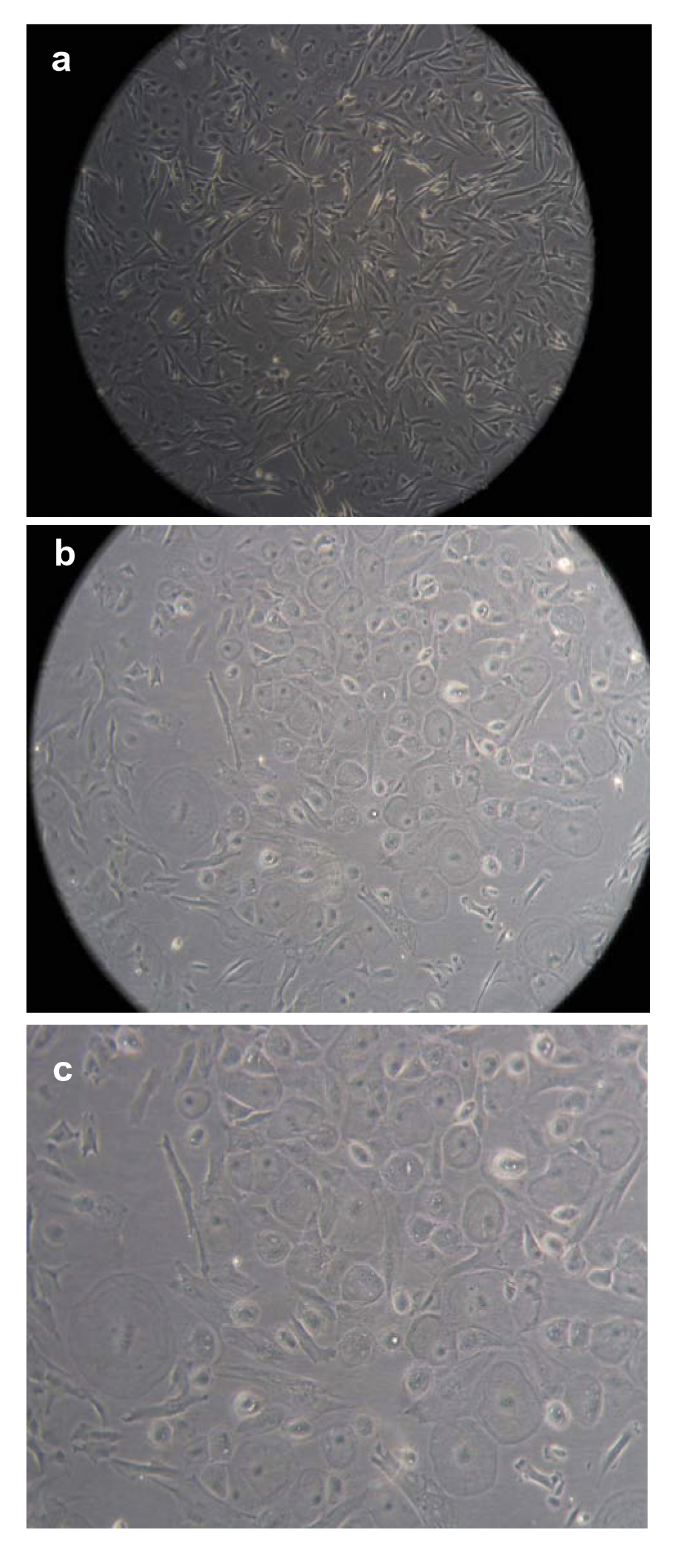

Supplement: Figure S2 — Effect of ICG-001 on cell morphology of hES cells in SAGM. H7 hES cells differentiated in SAGM were incubated with 5 µM ICG-001 in culture medium for 12 h as described in Figure 4 , and cell morphology was assessed a before, and b, c after incubation with ICG-001. a, b 10× and c 40× magnification. The data shown are representative of n = 3 independent experiments. (TIF) [file pone.0033165.s002.tif]

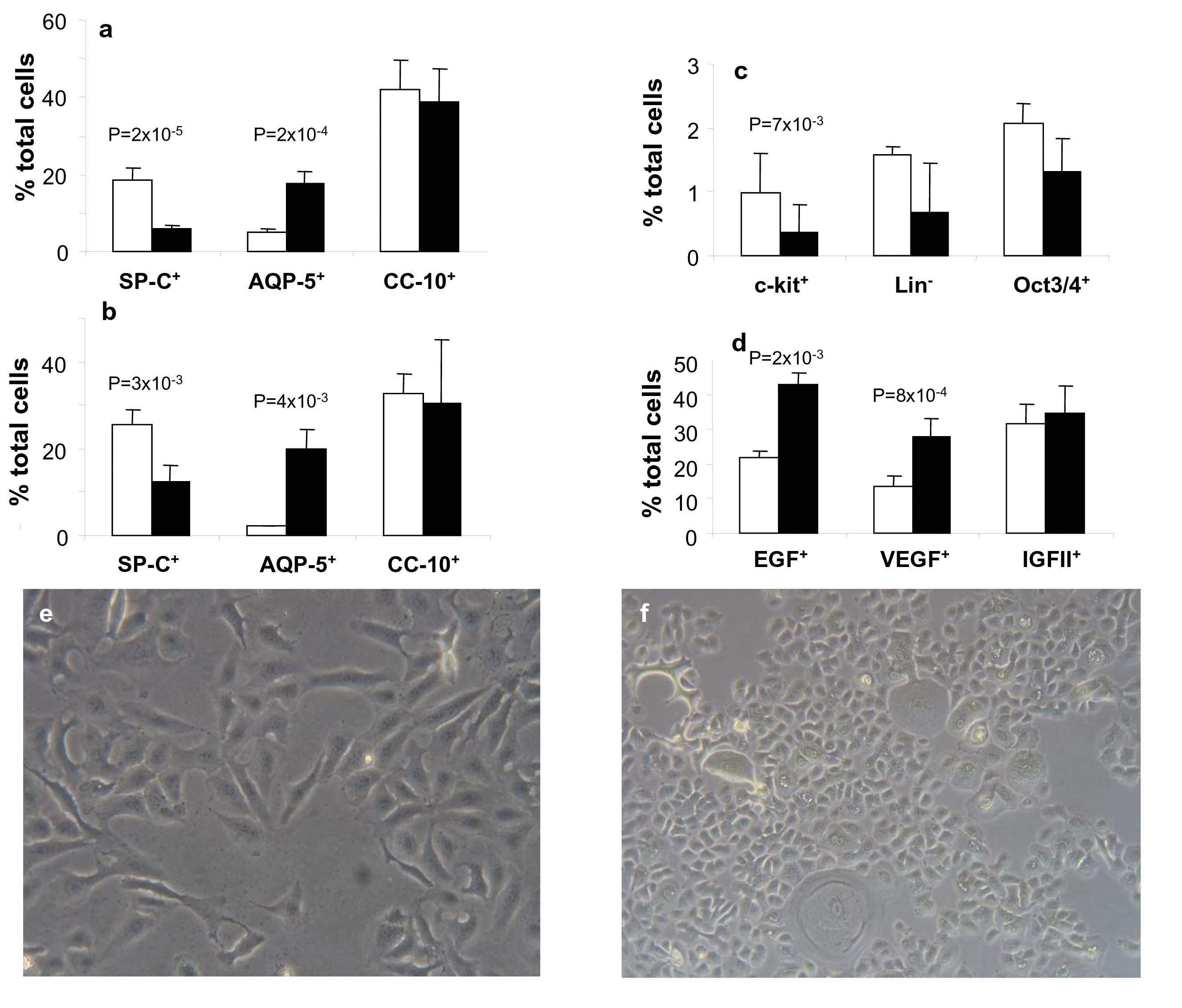

Supplement: Figure S3 — Effect of ICG-001 on hES cells differentiated in BEGM. Single cell suspensions of human H7 hES cells differentiated in BEGM and incubated in the a–d (open bars), e absence or a–d (solid bars), f presence of 5 µM ICG-001 in culture medium for 12 h underwent FACS to identify percentage by a intracellular and b surface expression of surface markers for AEII cells (SP-C+), AEI cells (AQP-5+), and Clara cells (CC-10+) and expression of c pluripotent markers (c-kit+, Lin−, Oct3/4+) and d growth factors (EGF+, VEGF+, IGFII+). The percent positive cells of total cells in culture are shown as mean ± SEM (triplicate sampling from three independent experiments). P<0.05 values in ICG-001-treated group vs. untreated group are shown. e, f 40× magnification. (TIF) [file pone.0033165.s003.tif]
