# Supplementary material for: Human Embryonic Stem Cells Differentiated to Lung Lineage-Specific Cells Ameliorate Pulmonary Fibrosis in a Xenograft Transplant Mouse Model
Source: PLoS One. 2012 Mar 28;7(3):e33165. doi: 10.1371/journal.pone.0033165 (PMC3314647; doi:10.1371/journal.pone.0033165)
Supplement: Table S1 — Mouse primers for qPCR. (DOCX) [file pone.0033165.s004.docx]

**Table S1. Mouse primers for qPCR.**

| **Marker** | **Gene** | **Forward primer** | **Reverse primer** |
| --- | --- | --- | --- |
| House- keeping | GAPDH | cgtcccgtagacaaaatggt | tcaatgaaggggtcgttgat |
|  | β-Actin | GTGGGCCGCTCTAGGCACCAA | CTCTTTGATGTCACGCACGATTTC |
| AEI cell | AQP-5 | AAGGGGTGATAGCCCTGTTT | GGCAATGTCCCCTCTGTCTA |
| AEII cell | SP-C | GCAAAGAGGTCCTGATGGAG | CTGGGACCTGCCGAGTAAT |
| Clara cell | CC-10 | CGCCATCACAATCACTGTG | TGACAAGCTTTAGCAGTAG |
| Growth factors | TGFβ_1_ | GGAGAGCCCTGGATACCAAC | CGCACACAGCAGTTCTTCTC |
|  | TGFβ_2_ | GGAGGTTTATAAAATCGACATGC | GGCATATGTAGAGGTGCCATC |
|  | TGFβ_3_ | TGGCTGTCTTTCGATGTCAC | TTTCCAGACCCAAGTTGGAC |
|  | VEGF-A | TACCTCCACCATGCCAAG | TGGTAGACATCCATGAACTTGA |
|  | VEGF− | GGCTTAGAGCTCAACCCAGA | TGGAAAGCAGCTTGTCACTTT |
|  | VEGF-C | GGGAAGAAGTTCCACCATCA | TCGCACACGGTCTTCTGTAA |
|  | FGF-1 | CAGCCTGCCAGTTCTTCAG | GGTTGTGATCTCCCCTTCAG |
|  | FGF-2 | AGAAGAGCGACCCACACG | TGGCACACACTCCCTTGATA |
| Collagen | Col 3α1 | AGGCCAGTGGCAATGTAAAG | CTCCATTCCCCAGTGTGTTT |
|  | Col 1α2 | GTCCTAGTCGATGGCTGCTC | CAATGTCCAGAGGTGCAATG |
|  | Col 6α1 | TGCCAAGGACTTCATCATCA | ACGTGCTCTTGCATCTGGTT |
