# Supplementary material for: Human Embryonic Stem Cells Differentiated to Lung Lineage-Specific Cells Ameliorate Pulmonary Fibrosis in a Xenograft Transplant Mouse Model
Source: PLoS One. 2012 Mar 28;7(3):e33165. doi: 10.1371/journal.pone.0033165 (PMC3314647; doi:10.1371/journal.pone.0033165)
Supplement: Table S2 — P<0.05 for Figure 2 : “Sequential downregulation of stem cell-specific surface proteins” panels e and f. Figure 2e data points where P<0.05. Figure 2f data points where P<0.05. (DOCX) [file pone.0033165.s005.docx]

**Table S2. P<0.05 for Figure 2: “Sequential downregulation of stem cell-specific surface proteins” panels e and f.**

***Figure 2e data points where P<0.05.***

| **Marker** | **Day 6** | **Day 7** | **Day 8** | **Day 9** | **Day 10** | **Day 11** | **Day 12** |
| --- | --- | --- | --- | --- | --- | --- | --- |
| TTF-1 | 0.003 | 0.004 | 0.020 | 0.003 | 0.038 | 0.003 | 0.010 |
| Oct3/4 | 0.004 | 0.005 | 0.013 | 0.043 | 0.003 | 0.008 | 0.009 |
| SSEA-3 | 0.003 | 0.003 | 0.013 | 0.069 | 0.008 | 0.010 | 0.010 |
| SSEA-4 | 0.003 | 0.003 | 0.026 | 0.001 | 2x10^-4^ | 0.004 | 0.030 |

***Figure 2f data points where P<0.05.***

| **Marker** | **Day 8** | **Day 9** | **Day 10** | **Day 11** | **Day 12** |
| --- | --- | --- | --- | --- | --- |
| TTF-1 | 0.002 | 0.004 | 0.012 | 0.032 | 0.043 |
| Oct3/4 | 0.004 | 0.002 | 0.004 | 0.043 | 0.046 |
| SSEA-3 | 0.008 | 0.002 | 0.004 | 0.044 | 0.016 |
| SSEA-4 | 0.002 | 0.001 | 0.011 | 0.044 | 4x10^-4^ |
