# Supplementary material for: Human Embryonic Stem Cells Differentiated to Lung Lineage-Specific Cells Ameliorate Pulmonary Fibrosis in a Xenograft Transplant Mouse Model
Source: PLoS One. 2012 Mar 28;7(3):e33165. doi: 10.1371/journal.pone.0033165 (PMC3314647; doi:10.1371/journal.pone.0033165)
Supplement: Table S3 — P<0.05 for Figure 3 : “Phenotypic analysis and ultrastructure of hES cells differentiated to lung epithelial cell-specific lineages” panels a, b, e, and f. Figure 3a marker data points where P<0.05. Figure 3b marker data points where P<0.05. Figure 3e marker data points where P<0.05. Figure 3f marker data points where P<0.05. NS, Not Significant. (DOCX) [file pone.0033165.s006.docx]

**Table S3. P<0.05 for Figure 3: “Phenotypic analysis and ultrastructure of hES cells differentiated to lung epithelial cell-specific lineages” panels a, b, e, and f.**

***Figure 3a marker data points where P<0.05.***

| **Marker** | **Day 4** | **Day 5** | **Day 6** | **Day 7** | **Day 8** | **Day 9** | **Day 10** | **Day 11** | **Day 12** |
| --- | --- | --- | --- | --- | --- | --- | --- | --- | --- |
| SP-C | 0.013 | 0.007 | 0.001 | 3x10^-7^ | 2x10^-6^ | 3x10^-5^ | 3x10^-9^ | 4x10^-5^ | 0.005 |

***Figure 3b marker data points where P<0.05.***

| **Marker** | **Day 4** | **Day 5** | **Day 6** | **Day 7** | **Day 8** | **Day 9** | **Day 10** | **Day 11** | **Day 12** |
| --- | --- | --- | --- | --- | --- | --- | --- | --- | --- |
| SP-C | 0.002 | 0.006 | 0.006 | 0.007 | 0.007 | 6x10^-4^ | 0.006 | 6x10^-4^ | 0.004 |

***Figure 3e marker data points where P<0.05.***

| **Marker** | **Day 4** | **Day 5** | **Day 6** | **Day 7** | **Day 8** | **Day 9** | **Day 10** | **Day 11** | **Day 12** |
| --- | --- | --- | --- | --- | --- | --- | --- | --- | --- |
| CC-10 | 2x10^-6^ | 3x10^-7^ | 5x10^-6^ | 5x10^-5^ | 9x10^-6^ | 5x10^-7^ | 4x10^-6^ | 5x10^-6^ | 6x10^-7^ |
| SP-C | NS | NS | NS | NS | 0.004 | 5x10^-4^ | 0.002 | 3x10^-4^ | 4x10^-4^ |

***Figure 3f marker data points where P<0.05.***

| **Marker** | **Day 4** | **Day 5** | **Day 6** | **Day 7** | **Day 8** | **Day 9** | **Day 10** | **Day 11** | **Day 12** |
| --- | --- | --- | --- | --- | --- | --- | --- | --- | --- |
| CC-10 | 3x10^-5^ | 3x10^-5^ | 5x10^-5^ | 4x10^-5^ | 2x10^-5^ | 2x10^-5^ | 3x10^-5^ | 3x10^-5^ | 6x10^-5^ |
| SP-C | NS | NS | NS | NS | NS | NS | 0.002 | 3x10^-4^ | 3x10^-4^ |

NS, Not Significant
